# Properties and abundance of overlapping genes in viruses

**DOI:** 10.1093/ve/veaa009

**Published:** 2020-02-13

**Authors:** Timothy E Schlub, Edward C Holmes

**Affiliations:** v1 Sydney School of Public Health, Faculty of Medicine and Health,The University of Sydney, NSW, 2006, Australia; v2 School of Life and Environmental Sciences and School of Medical Sciences, Marie Bashir Institute for Infectious Diseases and Biosecurity, The University of Sydney, Sydney, NSW 2006, Australia

**Keywords:** overlapping genes, overprinted genes, reference genomes, meta data

## Abstract

Overlapping genes are commonplace in viruses and play an important role in their function and evolution. However, aside from studies on specific groups of viruses, relatively little is known about the extent and nature of gene overlap and its determinants in viruses as a whole. Here, we present an extensive characterisation of gene overlap in viruses through an analysis of reference genomes present in the NCBI virus genome database. We find that over half the instances of gene overlap are very small, covering <10 nt, and 84 per cent are <50 nt in length. Despite this, 53 per cent of all viruses still contained a gene overlap of 50 nt or larger. We also investigate several predictors of gene overlap such as genome structure (single- and double-stranded RNA and DNA), virus family, genome length, and genome segmentation. This revealed that gene overlap occurs more frequently in DNA viruses than in RNA viruses, and more frequently in single-stranded viruses than in double-stranded viruses. Genome segmentation is also associated with gene overlap, particularly in single-stranded DNA viruses. Notably, we observed a large range of overlap frequencies across families of all genome types, suggesting that it is a common evolutionary trait that provides flexible genome structures in all virus families.

## 1. Introduction

Overlapping genes are important in viruses. Not only do they maximise the amount of genetic information that can be encoded into genomes of usually small size (Bozarth, Weiland, and Dreher 1992; Bransom et al. 1995; Belshaw, Pybus, and Rambaut 2007; Bates et al. 2015; Brandes and Linial 2016), but mutations that introduce a new open reading frame (ORF) into an older established gene allow the creation of a *de novo* gene without major genomic restructuring (Chirico, Vianelli, and Belshaw 2010; Cui, Schlub, and Holmes 2014; Cassan et al. 2016). Although many overlapping genes lack known function, there is evidence that overlapping genes are associated with virus transmission and disease severity (Gogarten, and Townsend 2005; Cui, Schlub, and Holmes 2014; Fernandes et al. 2016), regulating gene expression (Johnson and Chisholm 2004; Bates et al. 2015; Han et al. 2017), and providing a variety of other fitness advantages (Keese and Gibbs 1992; Keeling and Palmer 2008). Characterising the nature and frequency of gene overlap in viruses is therefore central to understanding key aspects of virus evolution, as well as for accurate genome annotation and the characterisation of new viruses discovered through metagenomics (Krakauer 2000).

Studies investigating the determinants of gene overlap have revealed a number of noteworthy properties, such as a negative association with gene overlap and genome size (Keese and Gibbs 1992), a negative correlation between gene overlap and the frequency of synonymous substitution (Low et al. 2019), and upper bounds on the frequency of gene overlap (Cassan et al. 2016). However, understanding the nature of gene overlap in viruses and quantifying the frequency of its occurrence is incomplete as most studies only deal with a selection of viruses within a viral family or a small subset of reference genomes. A larger-scale comparative study is therefore required.

Herein, we quantify the extent, frequency and pattern of overlapping genes in all reference viral genomes (*n* = 7450) available on the NCBI viral genome database. In particular, we aimed to identify those factors associated with the presence and abundance of gene overlap, including the type of virus (RNA or DNA) and form of genome structure, including single-stranded (ss), double-stranded (ds), positive (+) and negative (−) sense, or segmented.

## 2. Methods

### 2.1 Detection of gene overlap in reference genomes

Reference genomes for 7,450 viruses were downloaded from the NCBI reference genome database on 31 January 2019. From this, annotation and sequence data were imported into *R: A language and environment for statistical computing* version 3.6.1 (R Core Team 2019). The following information was pulled from each ‘gbk’ file within the reference database: filename and path—used, along with organism name, to identify different segments of viruses with segmented genomes (different segments were stored in different files); GenBank accession number; the nucleotide sequence length of the entire reference genome; the genome structure (e.g. ssDNA circular) as defined by the ‘Locus’ row; organism name; the coding region of the virus genome (cds); the protein product name for each coding region (/product); the codon start position for each cds (/codon_start); and the nucleotide sequence itself. This information was stored in an R ‘list’ with each item in this list corresponding to an individual ‘gbk’ file from the reference genome database export.

Gene overlap within a genome was defined as any two coding regions (annotated by ‘CDS’ in reference genomes) that share at least 1 nt in their CDS range. Gene overlap was detected programmatically by looping through each CDS, and conducting pairwise comparisons to all other CDS’s within the genome for non-empty intersections. The length of overlap, direction of overlap, and name of the CDS products for the two overlapping genes were recorded. When a CDS was annotated as the join of two or more separate regions of the genome (such as when a ribosomal frameshift occurred, or the annotation used for circular genomes), individual joins were treated as separate CDS’s, after which results (such as number of overlaps) were aggregated at the CDS level. Antisense overlap was detected when one CDS was not annotated with a ‘complement’ and the overlapping CDS was annotated with a ‘complement’. A database (in R RDS format) of all instances of gene overlaps in Supplementary File S1. This can be loaded into R using the command readRDS (filepath).

### 2.2 Statistical methods

Proportions of the presence/absence of gene overlap across Baltimore groups were compared with an exact binomial test using the *binom.test* function in *R*. When testing for proportion differences across multiple Baltimore groups (e.g. RNA vs. DNA, or segmented vs. not segmented) a mixed logistic regression was used with a random intercept and virus family grouping structure. That is, different reference genomes within a virus family were treated as repeated samples. The mixed logistic regression was performed in R using the function *glmer* from the package *lme4* (Rancurel et al. 2009). Confidence intervals for proportions were obtained using the binom.test() function in R. Viral genome structure groups were defined by the Baltimore classification: Group I (dsDNA), Group II (ssDNA), Group III (dsRNA), Group IV (+ssRNA), Group V (−ssRNA), Group VI (ssRNA-RT), and Group VII (dsDNA-RT).

## 3. Results

### 3.1 Total frequency of gene overlap across all viruses

**Figure 1.**
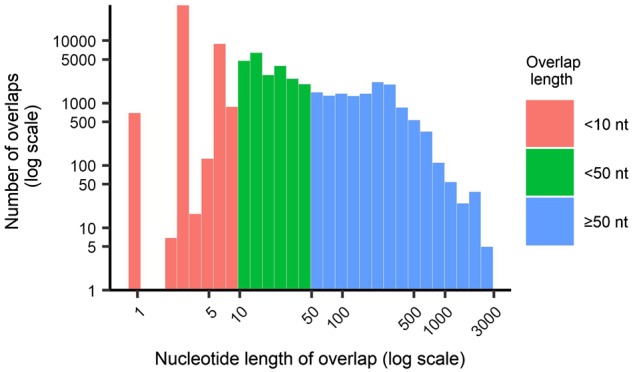
Logarithmic scaled histogram of the length of gene overlap. Overall, 54 per cent of gene overlaps are <10 nt in length, and 81 per cent of gene overlaps are <50 nt in length.

**Figure 2.**
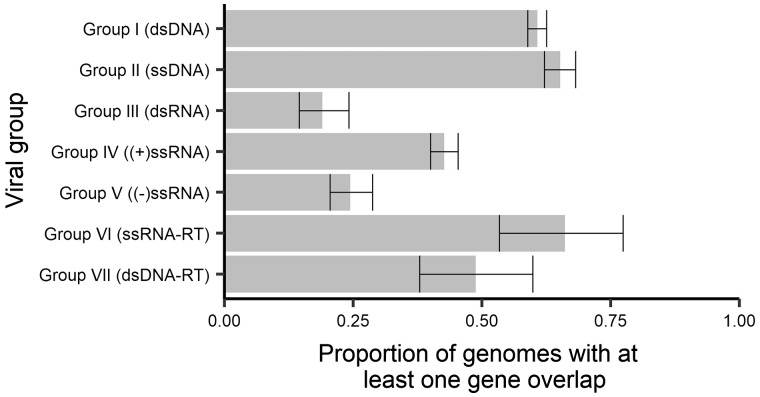
Proportions of genomes with at least one instance of gene overlap (>50 nt) across viral groups. Error bars represent 95 per cent CI for the proportion.

**Figure 3.**
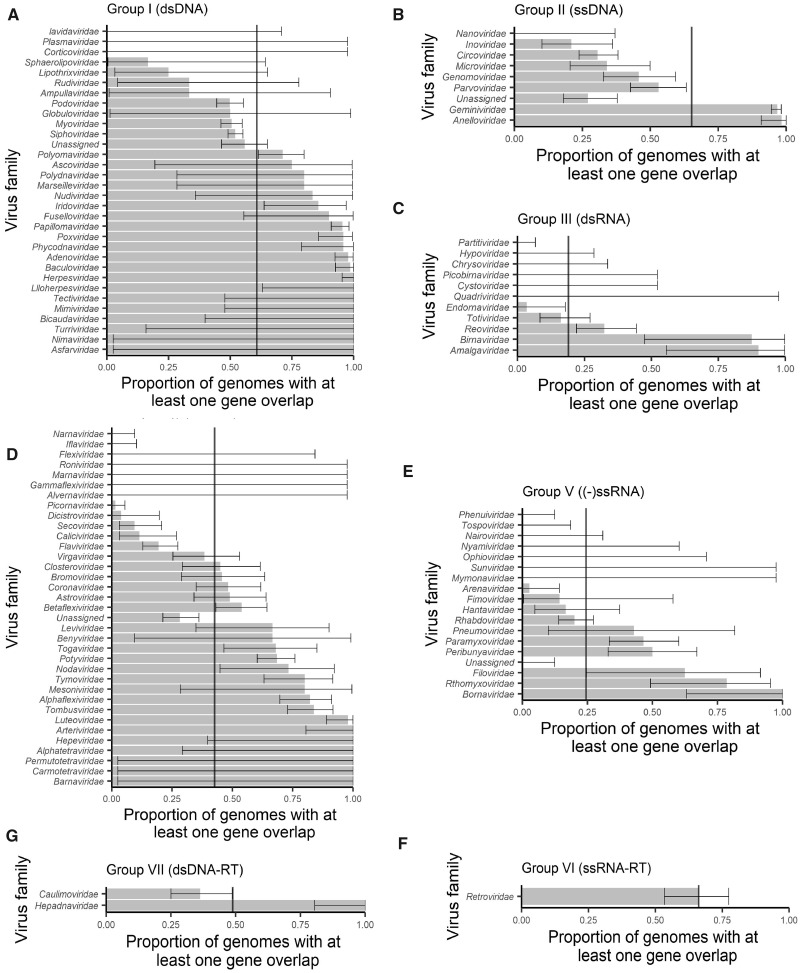
Proportions of genomes with at least one instance of gene overlap across viral groups, stratified by virus family. Virus families are ordered by their proportion and then the width of the confidence intervals. Error bars represent 95 per cent CIs for the proportion within a family. Vertical lines represent overall means within a viral genome group.

**Figure 4.**
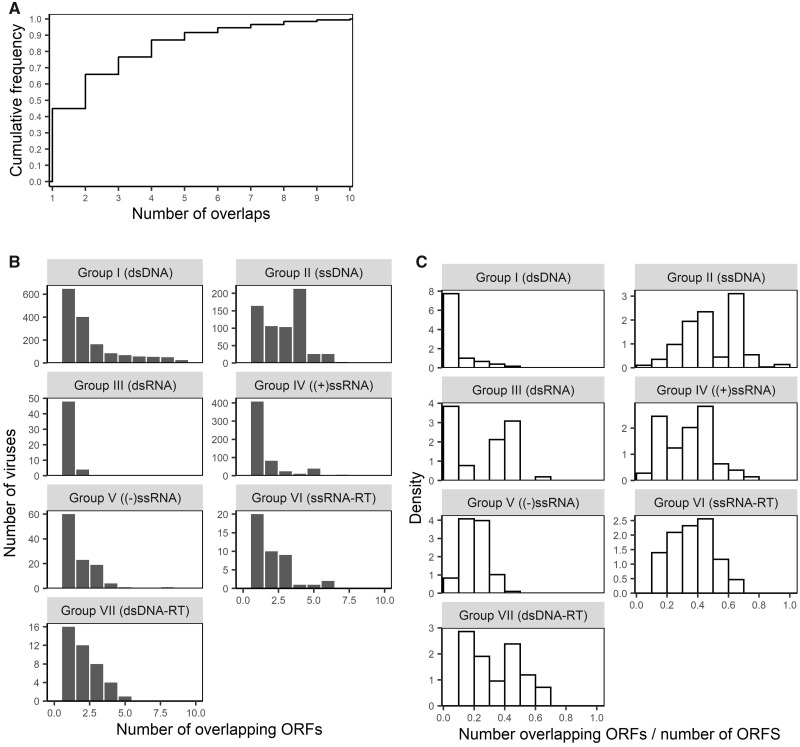
(A) The cumulative distribution of the total abundance of gene overlap (number of gene with an overlap per genome) over all viruses studied here. (B) Histograms of the total abundance of gene overlap by virus family, truncated at 10. All virus groups had a maximum total abundance less than or equal to 10, except dsDNA viruses with a very long tail up to 789, and ssDNA viruses with a maximum of 15. (C) Histograms of the total abundance of gene overlap by family as a proportion of the number of genes (relative frequency = number genes with an overlaps/number of genes).

**Figure 5.**
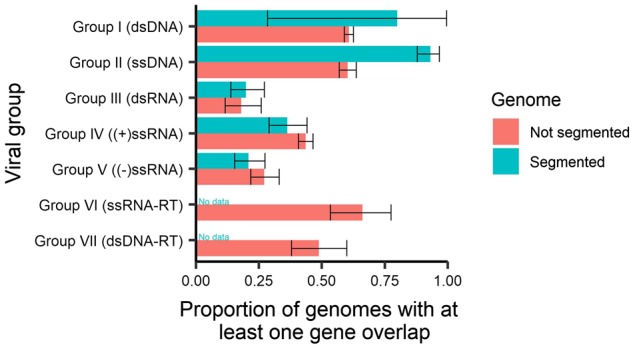
Proportion of genes with at least one instance of gene overlap stratified by segmented genomes. Error bars represent 95 per cent CIs.

**Figure 6.**
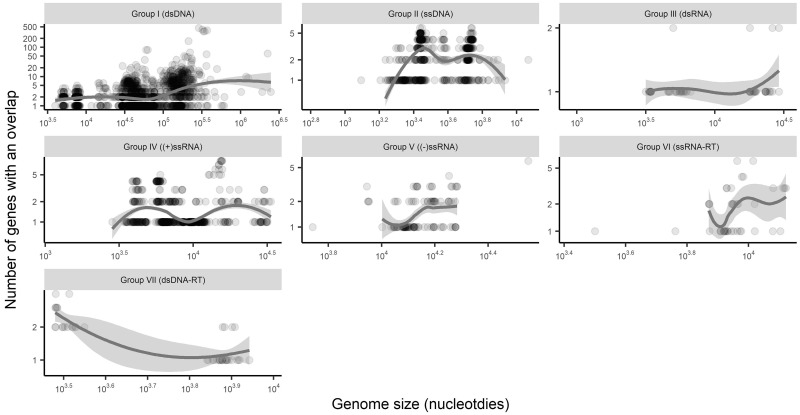
Number of genes involved in gene overlap by genome size (excluding all genomes with no overlap). Trend lines are Loess curves with span 0.80. Both the x and y axes depict log scales.

**Figure 7.**
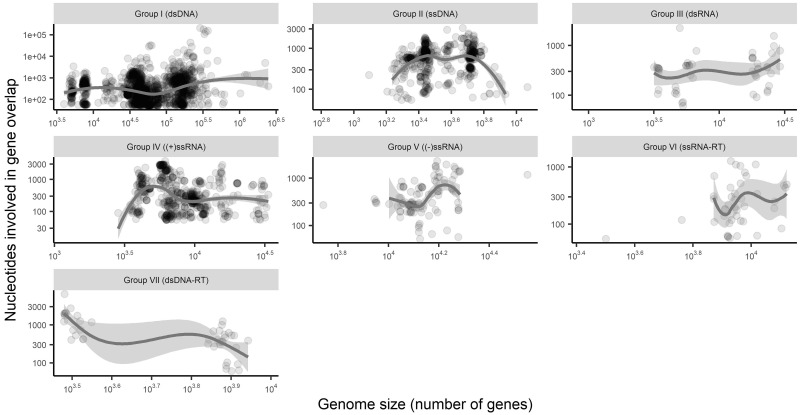
Total number of nucleotides involved in gene overlap by genome size (excluding all genomes with no overlap). Trend lines are Loess curves with span 0.80. Both the x and y axes depict log scales.

**Figure 8.**
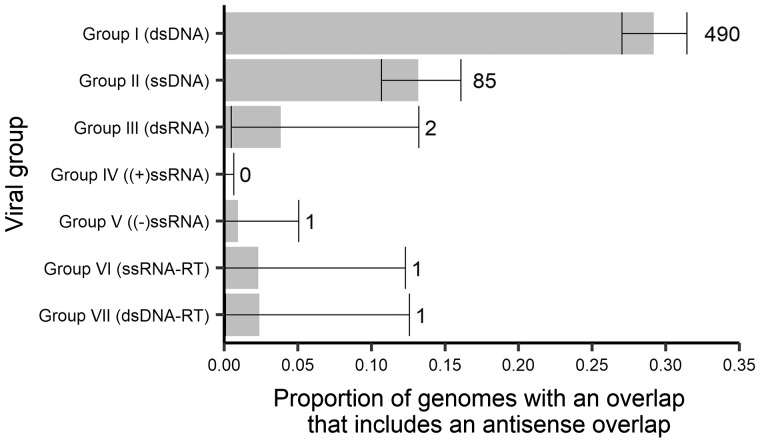
The proportion of genomes containing a gene overlap that has an antisense overlap in each virus group. Error bars represent 95 per cent CIs. Numbers to the right of error bars represent the number of genomes containing an antisense gene overlap (e.g. 490 genomes in Group I (dsDNA) contain an antisense gene overlap).

To quantify the extent of gene overlap in viruses we downloaded reference genomes for all viruses available on the NCBI virus reference database. Of the 7,450 reference genomes collected, 191 satellite virus genomes and 1,283 currently unclassified virus genomes were removed due to insufficient taxonomic information, leaving 5,976 reference genomes. Using this data collection, we identified all instances of overlapping coding regions excluding those occurring in the same reading frame—frequently a product of employing an alternative start codon. Accordingly, across the 5,976 reference genomes analysed, we identified 83,722 instances of reported gene overlap occurring across 127,940 coding regions (‘cds’ annotation in GenBank). Notably, however, 57 per cent (47,972) of these overlaps were very small in length, covering <10 nt, and 84 per cent (70,533) of instances were small relative to the length of complete coding sequences (<50 nt; Fig. 1). The most common of these small overlap lengths was 3 nt (44.5%) followed by 7 nt (9.6%), 10 nt (4.7%), and 13 nt (4.0%). As gene overlap of such limited lengths are less likely to impact genomic structure and virus evolution than larger overlaps, they were excluded from all subsequent analyses. This left 13,189 instances of gene overlap >50 nt in length that we studied in more detail. Hence, the results that follow are for these larger (>50 nt) overlaps only and these include instances of overlap with putative or hypothesised genes. In Supplementary Files S2 and S3, we show the equivalent results (i.e. as presented in Figs 2–8) for overlaps >10 and 100 nt in length, respectively. This resulted in similar comparative trends.

### 3.2 Presence and absence of gene overlap across viral groups and families

To describe the characteristics of gene overlap in all viruses, we first sought to determine what characteristics are associated with the presence/absence of at least one gene overlap. Of the 5,976 genomes studied, 53 per cent (3,175) contained at least one overlapping gene and this overall proportion varied among viral groups with different genome structures (i.e. as reflected in the Baltimore classification; Fig. 2). On average, we observe that after excluding retro-transcribing (RT) viruses, RNA viruses contained fewer reference genomes with gene overlap than DNA viruses, with gene overlap being least common in dsRNA viruses (19%, 95% CI 15–24%), followed by negative-sense ssRNA viruses (−ssRNA; 24%, 95% CI 20–29%) and then positive-sense ssRNA viruses (+ssRNA; 43%, 95% CI 40–45%). Across the remaining Baltimore groups, dsDNA-RT viruses (such as the *Hepadnaviridae*) had 49 per cent of genomes containing an overlap (95% CI 38–60%) dsDNA viruses (61%, 95% CI 59–63%), ssDNA viruses (65%, 95% CI 62–68%), and ssRNA-RT viruses (66%, 95% CI 53–77%).

Importantly, the trend of lower gene overlap in RNA (in non-RT groups) compared with DNA viruses remains when proportions are broken down by virus family (Fig. 3), indicating that this trend is not driven by unequal numbers of genera among families nor the correlations expected across genera within the same family. The results are further confirmed with a mixed regression model that accounts for correlated data within viral families (DNA to RNA comparison *P* < 0.001, OR = 9.76, 95% CI 3.47–30.76%; RNA groups comparison *P* < 0.001). However, despite these trends of average behaviour at the level of viral genome type, there still exists extensive diversity in the proportion of genomes with an overlap across families of the same genome type (ssDNA, dsDNA, −ssRNA, etc.). In fact, the proportion of viruses in a family with at least one gene overlap ranges from 0 to 100 per cent within most genome types excluding RT viruses for which there are very few families (Fig. 3). This indicates that the specific evolutionary history of individual viral families may play a far greater role in determining the presence of gene overlap than overall genome type.

### 3.3. Abundance of gene overlap across viral groups and families

In addition to the frequency of gene overlap, we examined the abundance of gene overlap within genomes that contained at least one overlapping gene. Here, abundance can be measured either as the total number of instances of gene overlap within a genome (total abundance), or the relative frequency of overlapping genes as measured by the proportion of genes within which they are overlapping (i.e. relative abundance = proportion of genes involved in overlap ÷ number of genes). Interestingly, most genomes with overlapping genes have only low total abundance, but most also have >1 overlap, with 43 per cent genomes having only 1 gene overlap, 64 per cent at most 2 overlaps, and 74 per cent at most 3 overlaps (Fig. 4A). Across the different viral groups, the median total abundance was at most 3, and the 90th percentile was <5 in all groups excluding dsDNA (Fig. 4B). Within dsDNA viruses, there was a long tail in the distribution of total abundance with overlapping genes common found in viruses from the family *Phycodnaviradae* that infect algae. The most extreme case of this was the *Acanthocystis turfacea chlorella* virus that contains 860 genes (many classified as putative or hypothetical), of which 459 were overlapping, creating 789 instances of gene overlap (as genes can overlap multiple genes). Although the total abundance was low across viral groups, relative abundance varied considerably across groups primarily due to differences in gene abundance (Fig. 4C) (the denominator of the relative abundance measure).

### 3.4 Gene overlap in segmented viruses

Segmented viruses exist in all non-RT virus groups, but are uncommon in dsDNA viruses (5 viruses, compared with 147–187 viruses in the ssDNA, dsRNA, +ssRNA, and −ssRNA groups). Segmented and non-segmented viruses had different proportions of gene overlap presence within most genome types. Segmented viruses were more likely to contain a gene overlap when they comprised ssDNA genomes (*P* < 0.001), marginally less likely in those with +ssRNA genomes (*P* = 0.07), and equally likely in dsRNA and −ssRNA viruses (*P* = 0.35 and 0.13, respectively; Fig. 5). There were insufficient segmented viruses in the dsDNA group to make a suitable comparison. When looking over all Baltimore groups combined, and after adjusting for viral families using a mixed model, segmented viruses were more likely to contain an overlapping ORF (*P* < 0.001, OR = 1.06) than non-segmented viruses and there was no evidence for effect modification by Baltimore group (*P* = 0.65).

### 3.5 Gene overlap is associated with genome size

The final predictor of gene overlap we investigated was total virus genome size. Accordingly, we first compared genome size to gene overlap presence/absence. Surprisingly, we initially found no association between genome size and gene overlap presence after adjusting for viral group (*P* = 0.96, no adjustment for viral families). However, such a relationship does appear after adjusting for within-family correlations, with the probability of an incidence of gene overlap increasing as genome size increases (*P* = 0.001, OR = 2.3 for every 10-fold increase in sequence size). With respect to overlap abundance, the number of genes that have an overlap (Fig. 6) and the number of nucleotides involved in gene overlap (Fig. 7) have varied and inconsistent relationships with genome size that depend on the viral group as defined by genome types. Importantly, this shows that the total variability in gene overlap abundance is in general poorly predicted by genome size. This is in part because, as noted above, most genomes have three or fewer instances of gene overlap, such that there is too little variability in total abundance to find consistent association patterns with other factors such as genome size. Additionally, as most genomes have three or fewer instances of gene overlap, relative abundance usually decreased with genome size.

### 3.6 Antisense gene overlap

Of those genomes that contained at least one overlapping gene that overlap generally occurs in the same sense as the remainder of the genome. However, antisense overlapping genes occurred in all virus groups with the exception of +ssRNA (Fig. 8), and ranged from small (50 nt, the cut-off) to large (2,351 nt) in length. Antisense overlaps were most frequent in DNA viruses. Within this group, 490 virus genomes across 26 families had antisense overlaps in dsDNA viruses, most of which occurred in the *Siphoviridae* (84 of 1,079 reference genomes), *Myoviridae* (82 of 544), *Baculoviridae* (72 of 73), *Herpesviridae* (65 of 76), *Poxviridae* (32 of 48), *Adenoviridae* (30 of 93), *Phycodnaviridae* (23 of 24), and *Podoviridae* (21 of 347). Overall, 85 genomes across six families had antisense overlaps in ssDNA viruses, but most of these (73) occurred in either the *Geminivridae* (52 of 423 reference genomes) or *Circoviridae* (21 of 170). There were only two instances of genomes with an antisense overlapping gene within dsRNA viruses, both in the *Reoviridae*, and one instance in −ssRNA viruses (*Arenaviridae*), ssRNA-RT viruses (hypothetical antisense gene in HIV1; Sabath, Wagner, and Karlin 2012), and dsNDA-RT viruses (*Caulimoviridae*).

### 3.7 Types of gene overlap

Gene overlap can occur when a gene exists totally within another gene, or when the termini of two genes overlap. Of the 13,189 instances of overlap, 30 per cent (3,979) occur totally within another gene. This proportion was similar when looking at dsDNA, ssDNA, and +ssRNA viruses (30.4, 26.3, and 31.3%, respectively), but higher in dsRNA and −ssRNA viruses (37.5 and 56.8%, respectively). Gene overlap can also encompass more than one other gene. The 13,189 instances of overlap studied here involve 20,499 genes. Of these genes, 78.6 per cent (16,103) overlap only one other gene, 16.9 per cent (3,474) overlap two other genes, 3.1 per cent (628) overlap three genes, and 1.4 per cent (293) overlap four or more genes. For the 293 cases with four or more overlaps, 208 (70.7%) are from the *Phycodnaviridae* which have very high levels of overlap abundance. However, the two viruses that contain genes with the largest number of overlaps were the Stx converting phage 1 and 2 viruses. These two viruses are dsDNA bacteriophages within the *Podoviridae* and both have a hypothetical gene that overlaps with 18 (17 for Stx converting phage 2 virus) other hypothetical genes.

### 3.8 Identical overlapping ORFs across genera and families

Finally, to investigate the potential inheritance of overlapping ORFs over evolutionary time, we identified overlapping ORFs of >10 nt in length that were present in multiple viruses from different genera or families. Such an overlap was defined as having 95 per cent similarity in nucleotide sequence (equivalent to 100% similarity for sequences of size 10–19 nt). Strikingly, all instances of shared overlap across families that were >25 nt occurred in the *Podoviridae* and *Siphoviridae*—two families of dsDNA bacteriophage (Supplementary File S4). The largest of these shared overlaps was 185 nt in length that occurred in bacteriophage that infect *Salmonella*, *Shigella*, and *Escherichia* genera bacteria. Across genera within a family, all gene overlaps >25 nt in length also occurred in dsDNA bacteriophages—in either the *Myoviridae* or *Microviridae* families across the *Escherichia*, *Shigella*, *Klebsiella*, *Citrobacter*, and *Salmonella* phages. The largest of these was 112 nt in length between *Escherichia* phage JS98 and *Enterobacteria* phage JS10. Smaller shared overlaps were also predominately in dsDNA bacteriophages present in the *Siphoviridae*, *Podoviridae*, and *Myoviridae* families. However, a small set of shared overlaps—either across or within families—between 11 and 25 nt in length was also apparent. Notably, only two instances of shared overlap occurred in non-DNA viruses. Both of these were +ssRNA viruses and were 1) an overlap of 22 nt between Vanilla latent virus and Alfalfa virus S in the *Alphaflexiviridae* family, and 2) an overlap of 18 nt between Ball python nidovirus 1 (*Coronaviridae*) and Morelia viridis nidovirus (unclassified family within the order *Nidovirales*) (Supplementary File S4).

## 4. Discussion

To our knowledge, this is the first attempt to reveal, in a quantitative manner, the extent and pattern of gene overlap among viruses of all genomic types, in doing so highlighting outstanding questions in this field. The importance of overlap size is one such question. The most frequent overlap observed was of only 4 nt—a single codon frameshifted by 1 nt. This itself is perhaps unsurprising given that the stop codon of TGA can form a Methionine (ATG) start codon if prefixed by an A and frameshifted by 1 nt. However, whether this or other small overlaps confer any meaningful selective advantage, or are just a convenient coincidence, is difficult to determine. One way to assess this is by examining overlaps that exist across genera or across viral families, such that they may have been selectively preserved. However, this analysis presents both computational and data challenges. Computationally, the number of small overlaps is extremely large (47,972) such that more than 2 billion (47,972^2^) pairwise comparisons would be needed to identify shared overlaps. Defining a shared overlap is also difficult due to the inconsistency in reference genome annotation. For example, a shared overlap would ideally be an identical (or near identical) nucleotide sequence of overlap of the same two genes across different viruses. Although sequence identity is straightforward to measure, gene identity can be difficult to determine with systematic rules due to different annotation practices: for example, the ‘/product’ annotation in the reference database would be the best candidate to identify genes but has a very inconsistent naming. Given these computational difficulties, we restricted our analysis to overlaps >10 nt and define shared overlap by sequence identity alone (using a 95% sequence identity cut-off to avoid data inconsistency difficulties). Although this choice of cut-off length is arbitrary, we note that the number of shared overlaps increases dramatically at 10 or fewer nt in length.

A number of possible mechanisms may explain the emergence of shared overlaps. First, shared overlaps could occur by chance alone. This mechanism is feasible for short overlaps but becomes increasingly unlikely as overlap length increases and must be deemed highly unlikely for the largest shared overlaps observed. Shared overlaps may also appear in viruses simply because they are inherited from a common ancestor, a mechanism that seems reasonable for overlaps that are shared across genera within a family. Although this could in theory explain the 185 nt overlap shared between members of the *Siphoviridae* and *Podoviridae*, as these families seem unlikely to be sister groups (Shcherbakov, and Garber 2000; Salzberg 2007) convergent evolution may be a more plausible explanation in this case. Indeed, it is possible that convergence may explain other overlaps that are distributed across wide phylogenetic distances. However, it is striking that the viruses predominantly involved in shared overlaps are bacteriophage that infect *Salmonella*, *Shigella*, and *Escherichia* genera bacteria. Importantly, these bacteria commonly experience horizontal gene transfer mediated by bacteriophage induced transduction, in which bacterial DNA is packaged into the bacteriophage genome through recombination and then transferred to a different bacterial cell (Simon-Loriere, Holmes, and Pagán 2013; Torresilla, Mesnard, and Barbeau 2015). Given the extent of shared overlap among these bacteriophages, it is possible that horizontal gene transfer is in part responsible for the high levels of shared gene overlap observed, although the detailed characterisation of these events will require additional analyses beyond the scope of this article.

Overlaps of at least 50 nt show a number of other interesting patterns across genome structure types and across families. In particular, DNA viruses harbour both a greater proportion of viruses with at least one gene overlap and have a higher prevalence of multiple overlaps within a virus compared with RNA viruses. However, despite these average trends across genome types, the trends across families show very high diversity in the prevalence of gene overlap. Importantly, this indicates that genome structure alone (e.g. DNA vs. RNA, or single- vs. double-stranded) does not provide good predictive power for assessing the likelihood of gene overlap. Furthermore, although some families show remarkable consistency in the prevalence of gene overlap (such as those with almost all or none with an overlap in Fig. 3, including the *Papillomaviridae* that almost always contain an overlap), some virus families are highly variable (such as those with a proportion closer to 0.5 in Fig. 3, including the *Myoviridae*, *Siphoviridae,* and *Podoviridae*). Another factor commonly associated with gene overlap is genome length (Keese and Gibbs 1992; Belshaw, Pybus, and Rambaut 2007), and although we do observe some weak association in some groups (Figs 6 and 7) in reality genome size explains little of the variability of gene overlap abundance, largely because most viruses have relatively few instances of gene overlap (Fig. 4A and B).

Another notable observation was the identification of antisense overlaps in both DNA and RNA viruses (Fig. 8), reflecting the case in which two genes that overlap that are coded in a direction antisense to each other. It is not surprising that most antisense overlaps occur in DNA virus families as these families also contain high levels of antisense genes. More surprising, however, was the presence of both antisense genes and overlaps in all groups of RNA viruses, excluding +ssRNA, even if this presence is very low with just one or two genomes in each of these groups (*Reoviridae* for dsRNA, *Arenaviridae* for −ssRNA, *Retroviridae* (HIV-1) for ssRNA-RT, and *Caulimoviridae* for dsRNA-RT).

A limitation of this study is the reliance on GenBank annotation, particularly for the nucleotide position that starts the coding sequence. For example, some GenBank annotations will begin at the first in frame start codon (ATG) even if the coding product does not truly begin here. Importantly, eliminating overlaps that are <50 nt in length, as we have done here, is likely to remove many instances of overlap due to incorrect start codon annotation. However, this will not improve overlap size estimation and so overlap sizes reported in this article will be smaller when coding regions are incorrectly annotated. Additionally, as with any large long-term curated database, entries will become outdated with time as knowledge and annotation practices improve (Zhang, Shi, and Holmes 2018).

In addition to revealing general trends, a key outcome of this study is the creation of a database of gene overlap in Supplementary File S1. This provides researchers with the resources required to identify families, genera or viruses, with high or low prevalence of overlapping genes. It can also be used to expand on the analysis presented here and investigate other associations using the information captured in the annotation of reference genomes. Overlapping genes are increasingly being demonstrated to play an important role in viral function and evolution. Understanding this impact requires careful quantification and visualisation of the extent of gene overlap, and the conditions in which overlap occurs and persists as we have done here.

## Funding

EH is an ARC Australian Laureate Fellow (FL170100022).

**Conflict of interest:** None declared.

## Supplementary Material

veaa009_Supplementary_Data
