# Supplementary material for: Properties and abundance of overlapping genes in viruses
Source: Virus Evol. 2020 Feb 13;6(1):veaa009. doi: 10.1093/ve/veaa009 (PMC7017920; doi:10.1093/ve/veaa009)
Supplement: veaa009_Supplementary_Data [file veaa009_supplementary_data.zip › Figure legends for supplemtary figures.docx]

**Figure Legends – Supplementary File 2 (10nt cut-off)**

**Figure 2**. Proportions of genomes with at least one instance of gene overlap across viral groups. Error bars represent 95% confidence intervals for the proportion. Equivalent to figure 2 in the main text but for all overlaps of size >10nt instead of the 50nt cut-off in the main text.

**Figure 3**. Proportions of genomes with at least one instance of gene overlap across viral groups, stratified by virus family. Virus families are ordered by their proportion and then the width of the confidence intervals. Error bars represent 95% confidence intervals for the proportion within a family. Vertical lines represent overall means within a viral genome group. Equivalent to figure 3 in the main text but for all overlaps of size >10nt instead of the 50nt cut-off in the main text.

**Figure 4**. (A) The cumulative distribution of the total abundance of gene overlap (number of gene with an overlap per genome) over all viruses studied here. (B) Histograms of the total abundance of gene overlap by virus family, truncated at 10. All virus groups had a maximum total abundance less than or equal to 10, except dsDNA viruses with a very long tail up to 789, and ssDNA viruses with a maximum of 15. (C) Histograms of the total abundance of gene overlap by family as a proportion of the number of genes (relative frequency = number genes with an overlaps / number of genes). Equivalent to figure 4 in the main text but for all overlaps of size >10nt instead of the 50nt cut-off in the main text.

**Figure 5**. Proportion of genes with at least one instance of gene overlap stratified by segmented genomes. Error bars represent 95% confidence intervals. Equivalent to figure 5 in the main text but for all overlaps of size >10nt instead of the 50nt cut-off in the main text.

**Figure 6**. Number of genes involved in gene overlap by genome size (excluding all genomes with no overlap). Trend lines are Loess curves with span 0.80. Both the x and y axes depict log scales. Equivalent to figure 6 in the main text but for all overlaps of size >10nt instead of the 50nt cut-off in the main text.

**Figure 7**. Total number of nucleotides involved in gene overlap by genome size (excluding all genomes with no overlap). Trend lines are Loess curves with span 0.80. Both the x and y axes depict log scales. Equivalent to figure 7 in the main text but for all overlaps of size >10nt instead of the 50nt cut-off in the main text.

**Figure 8**. The proportion of genomes containing a gene overlap that have an antisense overlap in each virus group. Error bars represent 95% confidence intervals. Numbers to the right of error bars represent the number of genomes containing an antisense gene overlap (e.g. 490 genomes in Group I (dsDNA) contain an antisense gene overlap). Equivalent to figure 8 in the main text but for all overlaps of size >10nt instead of the 50nt cut-off in the main text.

**Figure Legends – Supplementary File 3 (100nt cut-off)**

**Figure 2**. Proportions of genomes with at least one instance of gene overlap across viral groups. Error bars represent 95% confidence intervals for the proportion. Equivalent to figure 2 in the main text but for all overlaps of size >100nt instead of the 50nt cut-off in the main text.

**Figure 3**. Proportions of genomes with at least one instance of gene overlap across viral groups, stratified by virus family. Virus families are ordered by their proportion and then the width of the confidence intervals. Error bars represent 95% confidence intervals for the proportion within a family. Vertical lines represent overall means within a viral genome group. Equivalent to figure 3 in the main text but for all overlaps of size >100nt instead of the 50nt cut-off in the main text.

**Figure 4**. (A) The cumulative distribution of the total abundance of gene overlap (number of gene with an overlap per genome) over all viruses studied here. (B) Histograms of the total abundance of gene overlap by virus family, truncated at 10. All virus groups had a maximum total abundance less than or equal to 10, except dsDNA viruses with a very long tail up to 789, and ssDNA viruses with a maximum of 15. (C) Histograms of the total abundance of gene overlap by family as a proportion of the number of genes (relative frequency = number genes with an overlaps / number of genes). Equivalent to figure 4 in the main text but for all overlaps of size >100nt instead of the 50nt cut-off in the main text.

**Figure 5**. Proportion of genes with at least one instance of gene overlap stratified by segmented genomes. Error bars represent 95% confidence intervals. Equivalent to figure 5 in the main text but for all overlaps of size >100nt instead of the 50nt cut-off in the main text.

**Figure 6**. Number of genes involved in gene overlap by genome size (excluding all genomes with no overlap). Trend lines are Loess curves with span 0.80. Both the x and y axes depict log scales. Equivalent to figure 6 in the main text but for all overlaps of size >100nt instead of the 50nt cut-off in the main text.

**Figure 7**. Total number of nucleotides involved in gene overlap by genome size (excluding all genomes with no overlap). Trend lines are Loess curves with span 0.80. Both the x and y axes depict log scales. Equivalent to figure 7 in the main text but for all overlaps of size >100nt instead of the 50nt cut-off in the main text.

**Figure 8**. The proportion of genomes containing a gene overlap that have an antisense overlap in each virus group. Error bars represent 95% confidence intervals. Numbers to the right of error bars represent the number of genomes containing an antisense gene overlap (e.g. 490 genomes in Group I (dsDNA) contain an antisense gene overlap). Equivalent to figure 8 in the main text but for all overlaps of size >100nt instead of the 50nt cut-off in the main text.
